# Proteostasis and resilience in the mechanically-stressed vascular endothelium

**DOI:** 10.1016/j.cophys.2023.100673

**Published:** 2023-08

**Authors:** Adam Keen, Feiran Zhang, John S Reader, Ellie Tzima

**Affiliations:** Wellcome Centre for Human Genetics, Radcliffe Department of Medicine, University of Oxford, United Kingdom

## Abstract

Endothelial homeostasis is a central feature of vascular health. The vascular endothelium is under constant mechanical stress resulting from blood flow and, therefore, requires a high degree of resilience to adapt to stresses and resist development of disease. In this review, we discuss the molecular mechanisms by which the endothelium maintains proteostasis in response to haemodynamic forces by regulating three key areas: protein synthesis, recycling and degradation.

**Current Opinion in Physiology** 2023, **34**:100673

This review comes from a themed issue on **Endothelium**

Edited by **Jeremy Pearson** and **Paul C Evans**

For complete overview of the section, please refer to the article collection, “Endothelium”

https://doi.org/10.1016/j.cophys.2023.100673

2468–8673/© 2023 The Author(s). Published by Elsevier Ltd. This is an open access article under the CC BY license (http://creativecommons.org/licenses/by/4.0/).

## Proteostasis in mechanically stressed endothelium

Protein homeostasis or proteostasis, is a key process by which cells maintain proper function and viability [1] that requires tight regulation through robust spatio-temporal control of sophisticated mechanisms that involve synthesis, degradation and recycling. The continuous exposure to environmental stresses makes maintenance of proteome balance a challenging task. Endothelial cells (ECs) line the inner surface of blood vessels where they are in direct contact with blood and bear the burden of the associated mechanical forces. Fluid shear stress (FSS) is the frictional force per unit area imposed on the endothelium due to the frictional force of the flowing blood and is a critical determinant of endothelial (dys)function [2]. Laminar shear stress (LSS) provides an atheroprotective environment where ECs maintain homeostasis. However, inflammatory signalling associated with low or oscillatory shear stress (OSS) at arterial bifurcations creates an atheroprone environment where atherosclerotic plaques develop [3].

**Figure 1:**
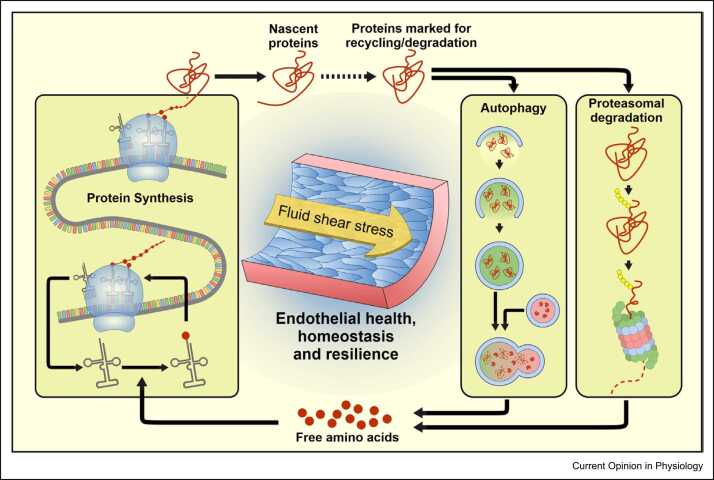
Proteostasis and resilience in the mechanically-stressed endothelium. ECs are subjected to FSS due to blood flow. Mechanical forces regulate proteostasis in ECs via control of protein synthesis, autophagy and proteasomal degradation.

To maintain the structural and functional homeostasis of blood vessels, ECs have developed a tightly regulated proteostasis network that ensures protein synthesis at the correct cellular location and time, recycling of cellular components by autophagy, and degradation of damaged proteins by the proteasome (Figure 1). The constant mechanical stress of flowing blood requires that the endothelium has evolved finely-tuned mechanisms to maintain proteostasis, which when awry, can lead to vascular pathologies, including endothelial inflammatory activation and atherosclerosis.

## Protein synthesis

A key component of the proteostatic network is protein synthesis. Although the effects of FSS on transcription have been the subject of several excellent reviews, how forces regulate protein production post-transcriptionally by mRNA translation is less well-understood 4, 5. A classic study by the Ingber group showed that application of mechanical stress to integrins using magnetic twisting forces resulted in localised mRNA and ribosomal recruitment, which is hypothesised to be indicative of mRNA translation [6]. Subsequent studies using application of FSS on ECs showed that this mechanical stimulus can regulate expression of E-selectin independent of changes to its mRNA. Specifically, FSS reduces binding of E-selectin mRNAs to actively translating polysomes and thus decreases protein levels, while simultaneously increasing global protein synthesis 7, 8. The identity of the mRNAs bound to polysomes in response to FSS remains to be identified.

A handful of studies have focused on the effects of FSS on the highly conserved mechanistic target of rapamycin (mTOR) pathway, which is a central regulator of protein synthesis [9]. mTOR is an atypical serine/threonine protein kinase that interacts with numerous proteins to form two distinct complexes: mTORC1 and mTORC2, distinguished by their sensitivity to rapamycin [9]. Both complexes contain mTOR and mLST8, however, Raptor and Rictor are unique to mTORC1 and mTORC2, respectively. Commonly, mTORC1 signalling is associated with protein translation, cell growth and proliferation, whereas mTORC2 is associated with regulation of cell survival and cytoskeletal organisation [9]. There is limited study on how FSS regulates the mTOR pathway. Kraiss et al. demonstrated that FSS activates p70S6K, the downstream target of mTOR [7], while another group reported that OSS causes sustained activation of mTOR/p70S6K and subsequent changes in protein expression via Smad1/5 10, 11. An elegant study by the Shyy group investigated the differential effects of LSS versus OSS on mTOR/p70S6K, demonstrating that while both LSS and OSS activate this pathway, the effects of LSS are transient, whereas activation persists under OSS [12]. In concordance, mTOR is also activated by low shear stress, as revealed by increased phosphorylation of its downstream target 4EBP1 [13].

Unsurprisingly, the mTOR pathway plays a critical role in endothelial homoeostasis, demonstrated using both cell-based and genetically modified mouse models. Inhibition of mTORC1 signalling is linked with hypertension and reduced vasodilation associated with endothelial dysfunction 14, 15, 16•. Critically, expression of a constitutively active form of S6K restores the endothelial dysfunction associated with loss of mTORC1, suggesting that this is, at least in part, due to restoration of protein synthesis [15]. In support of this model, endothelial-specific deletion of *Mtor*, *Raptor* or *Rictor* in mice causes defects in endothelial-dependent vasodilation due to impaired p70S6K-mediated post-transcriptional regulation of KLF2 expression [17]. Thus, although it is not clear if the effects are solely due to protein synthesis mechanisms, correct regulation of the mTOR pathway is required for EC homoeostasis.

## Autophagy

Another critical process that maintains proteostasis is autophagy: a specialised catabolic pathway that allows breakdown and recycling of intracellular components 18, 19. Although a multistep process that involves autophagy-related genes, it can be summarised as the formation of double-membrane vesicles, called autophagosomes, that degrade their contents after fusion with the lysosome and release into the cytoplasm for recycling [18]. Autophagy is triggered in response to different cellular stresses, including nutrient starvation, oxidative stress, hypoxia and infection [19]. In the cardiovascular system, autophagy is activated during ischaemia/reperfusion, heart failure and atrial fibrillation, but whether this represents a protective or deleterious response remains to be elucidated 19, 20, 21, 22.

Autophagy is intimately linked to endothelial function. Early studies showed that impairment of autophagy in ECs causes oxidative stress and impaired nitric oxide (NO) bioavailability [23], while autophagy-enhancing agents ameliorate EC dysfunction associated with ageing 24, 25. Several studies have reported that FSS regulates autophagy, which is generally associated with beneficial and atheroprotective effects 23, 26, 27, 28, 29, 30, 31, 32. Under LSS, endothelial nictric oxide synthase (eNOS) phosphorylation and consequent NO production are reduced in autophagy-deficient ECs, whereas reactive oxygen species and inflammatory cytokine production are increased 23, 26. In agreement, in an *ex vivo* perfusion model of LSS, autophagy inhibition reduced eNOS and increased endothelin-1 (ET-1) expression [26]. Interestingly, the eNOS-interacting protein caveolin-1 is a key regulator of autophagy, vascular inflammation and atherosclerosis [33], suggesting that autophagy regulates NO bioavailability through modulation of eNOS uncoupling [34]. More recently, it was shown that increased shear rate due to exercise is associated with increased indices of autophagy [32] and indices of autophagy and NO generation are present in radial artery ECs obtained from adult, but not older, volunteers [35]. A recent study linked disturbed flow with reduced autophagic flux, increased endothelial apoptosis and inflammatory activation via EVA1A [36]. Perhaps, the most convincing evidence for a causative role for autophagy in vessel atheroprotection comes from *in vivo* studies showing that genetic inactivation of endothelial autophagy increases murine atherosclerosis in high FSS areas that are normally atheroresistant 13••, 37. Defective autophagy is associated with increased EC apoptosis and senescence, coupled with defects in EC alignment in the direction of blood flow — a hallmark of EC health [13]. ECs defective in autophagy display increased inflammatory gene expression, decreasing expression of the anti-inflammatory factor KLF2, and increased monocyte adhesion 13••, 38.

In this context, autophagy is atheroprotective, promoting homeostasis by reducing inflammation and apoptosis 13••, 27, 38. However, as atherosclerotic disease progresses, autophagy could be viewed as either beneficial or detrimental. Autophagy promotes EC survival by degrading damaged intracellular components, reducing oxidative stress and protecting cells from apoptosis, but this also stabilises atherosclerotic plaques [39]. Autophagy suppresses the inflammasome, which in the short-term is an atheroprotective measure, however, as disease progresses, autophagy becomes defective, leading to increased plaque formation [40]. Excessive autophagy in ECs can also contribute to cell death and sustained ER stress in atherosclerotic lesions and downstream thrombosis [41]. In summary, autophagy is regulated by haemodynamic forces in ECs and its tight regulation is critical for endothelial homeostasis.

## Ubiquitin–proteasome system

Regulated degradation of proteins via the ubiquitin–proteasome system (UPS) ensures removal of damaged metabolites or misfolded proteins and, therefore, constitutes an important homeostatic mechanism [42]. Briefly, the UPS works in 2 stages: i) ubiquitination of target proteins, a post-translational modification that tags proteins with ubiquitin or ubiquitin-like proteins; and ii) degradation by 26S proteasome. The UPS cooperates with chaperones, HSP70 and HSP90, and chaperone cofactors in recognising misfolded proteins and targeting them for degradation.

Although incompletely understood, available evidence suggests that the UPS is a modulator of endothelial function [43]. For instance, NO mediates expression of immunoproteasome subunits and increased proteasome activity that could account for some of its cytoprotective effects [44]. Conversely, decreased NO is associated with reduced proteasome activity [45]. However, the relationship between the proteasome pathway and oxidative stress is rather complex, as oxidative stress can also promote formation of immunoproteasomes, degradation of damaged proteins and increased cell viability in murine inflammation models [46]. In this context, the UPS represents a homestatic response.

How FSS regulates the UPS is underexplored. Early studies showed that transcription of heat-shock proteins is increased after prolonged exposure to LSS 47, 48, suggestive of a role for FSS in activating the UPS. In agreement, proteomics analyses revealed that LSS increases the expression of the E1-like ubiquitin-activating enzyme responsible for covalent coupling of ubiquitin to target proteins, while it reduced the expression of the deubiquitination enzyme carboxyl-terminal hydrolase [49]. Consequently, LSS increased the number of ubiquitinated proteins [48]. The increase in expression of heat-shock proteins, coupled with increased ubiquitination observed under LSS, is presumably part of the homeostatic control mechanisms that involve proper folding and protein turnover. Although not tested at a global level, ubiquitination and proteasomal degradation can also be induced by OSS; the E3 ubiquitin ligase APC/Cdh1 interacts with the mechanosensor PECAM-1 and promotes its ubiquitination and proteasomal degradation. Application of OSS to ECs decreased expression of Cdh1, thereby stabilising PECAM-1 levels and increasing its expression. It was hypothesised that the induction of PECAM-1 protein level in ECs under OSS contributes to their inflammatory activation [50]. Whether this pathway is also responsible for regulation of ubiquitination of other target proteins in response to OSS remains to be determined. More recently, it was shown that shear stress stimulation of skeletal muscle ECs induces phosphorylation of the E3 ubiquitin ligase murine double minute-2, ubiquitination and proteasomal degradation of the forkhead transcription factor FoxO1 and downstream repression of angiogenesis [51].

A number of studies support a role for the UPS in endothelial inflammation and atherosclerosis (reviewed in Ref. [52]). The NO and NFκB pathways are amongst the best characterised. Briefly, proteasome inhibition modulates expression of eNOS, availability of its cofactors and its uncoupling [43]. Furthermore, ubiquitination leads to degradation of IκBα, which normally blocks nuclear localisation of NFκB, and leads to increased expression of cell adhesion molecules [53] and ET-1 [54]. However, ubiquitination is not only central to activation of inflammation, but can also terminate the inflammatory response by degradation of promoter-bound p50/p65 [55]. Additionally, ubiquitin-dependent degradation of programmed cell death 4 in response to atheroprotective shear stress, is important in reducing plaque burden in mice [56].

Several studies provide evidence for dysfunction of the UPS in atherosclerosis (reviewed in Ref. [52]), which has led researchers to consider atherosclerosis as a protein-quality disease [57]. The general consensus is that while enhanced proteasomal activity is observed in early atherosclerosis, which increases the inflammatory response (via NFκB and NO regulation), advanced atherosclerosis is characterised by impaired proteasomal activity and accumulation of dysfunctional proteins [52]. These observations are particularly pertinent when considering targeting of UPS function in atherosclerosis, as studies have yielded opposing results. Activity of the UPS declines with advanced atherosclerosis, as lesions from patients with stroke or transient ischaemic attack display lower proteasomal activity when compared with asymptomatic patients [58]. As atherosclerosis progresses, UPS appears to become dysregulated 57, 59, 60. Studies using inhibitors of the UPS have given mixed results that depend on the dosage of inhibitor used and stage of intervention, thus underscoring the complex role of the UPS in regulation of EC function [52].

## Perspectives

Correct function of the endothelium relies on tight regulation of factors affecting proteostasis. Owing to the constant mechanical stresses endured by the vascular endothelium, tight regulation of the molecular mechanisms that maintain proteostasis is necessary, as dysregulation at any stage from protein production, recycling or degradation is intimately linked to endothelial dysfunction and onset of disease (Figure 1). Further elucidation of proteostasis mechanisms will enhance our understanding of the homoeostatic and pathophysiological processes that underpin endothelial dysfunction and vascular disease.

## Declaration of Competing Interest

No competing interests are declared.

## Data Availability

No data were used for the research described in the article.
